# Health Care in Gulf Cooperation Council Countries: A Review of Challenges and Opportunities

**DOI:** 10.7759/cureus.1586

**Published:** 2017-08-21

**Authors:** Tawfiq Khoja, Salman Rawaf, Waris Qidwai, David Rawaf, Kashmira Nanji, Aisha Hamad

**Affiliations:** 1 Director General, Executive Board, Health Ministers’ Council for Cooperation Council States; 2 Department of Primary Care and Public Health, School of Public Health, Faculty of Medicine, Imperial College London, Uk; 3 Family Medicine, The Aga Khan University

**Keywords:** gulf cooperation council states, health care, health status, primary health care, health systems, migrants, migrant workers, health care needs

## Abstract

This study was undertaken to review the health care status in the Gulf Cooperation Council (GCC) member states, and explore current challenges and future opportunities. Available data was acquired using databases including PubMed, Embase, and Cochrane Library. The data gathered was then combined and the expert authors in the field discussed and propose strategies to overcome the challenges. There is an increase in both population and health care needs of GCC States citizens and migrant workers. The huge emigrant population challenges the capability of the already limited available health care resources. The region is faced with a quadruple disease burden that includes communicable and non-communicable diseases, mental health issues and accidental injuries. Recent advances in technology have made breakthroughs in diagnosis and treatment modalities but with an increase in overall health care cost. Innovative and cost-effective strategies are required to cater the health care needs of people living in the GCC states. Policy makers should emphasize the need to prioritize and strengthen primary care as a matter of urgency.

## Introduction and background

The Gulf Cooperation Council (GCC) consists of six countries, Bahrain, Kuwait, Oman, Qatar, Saudi Arabia, and United Arab Emirates (UAE), as member states. These countries are currently experiencing an increased demand for health care services due to an immense population growth, increasing life expectancy and higher incidence of non-communicable diseases (NCDs). There is a need to develop strategies to overcome these challenges [1-2].

Significant investments in health care infrastructure by GCC governments were observed in the past 25 years in the form of large medical cities and complexes. This increase in hospitals and clinics raised the quality of healthcare services in the region [2]. According to WHO World Health Statistics 2014, the life expectancy in GCC countries has increased to 77 years and infant mortality is decreased to 8 deaths per 1,000 live births in 2013 [2]. The overall number of doctors increased to 9.1% in 2012, averaging 2.5 doctors per 1000 population and general practitioners are on an average 2.1 per 1000 population [3]. Nursing personnel increased in the same period by 7.3%, averaging 4.7 nursing staff per 1000 population. Around 13,000 new hospital beds were added in the GCC between 2009 and 2013, though the number of hospital beds per 1000 population stood at 1.9 in 2013 [2].

Health care expenditure in GCC is less than the expenditure incurred in the developed countries, though the income levels are generally comparable. GCC healthcare services cost USD 18 million in 2008 and are expected to rise to USD 69.4 Billion by 2018. Health care costs are projected to increase more for outpatient departments (OPD). In 2013, outpatient visits accounted for about 65% of all visits to the hospitals [1,3].

The rising health care costs are largely related to the absence of a specialty, or the quality of the available treatment in the home country. According to a poll, about 39% UAE nationals said they would travel abroad for treatment, 47% in Bahrain and 43% in Qatar and Oman would also prefer to get treated abroad [3-4]. Even though a higher proportion of patients choose to travel abroad, the Gulf residents reported that they are satisfied with the quality of healthcare services provided. Treatment costs incurred abroad are mostly paid by the health care authorities and other government agencies in the Council States. The health services in GCC are provided free of cost to all residents and health insurance is available for both the expatriates and nationals. However, to meet the growing demands of the healthcare delivery system in GCC there is a need of a strong and capable health care delivery system.

This paper reports a pragmatic analysis of the current state and challenges of health care delivery system in GCC countries and possible strategies to overcome these issues.

## Review

Overview of healthcare in GCC

**Figure 1:**
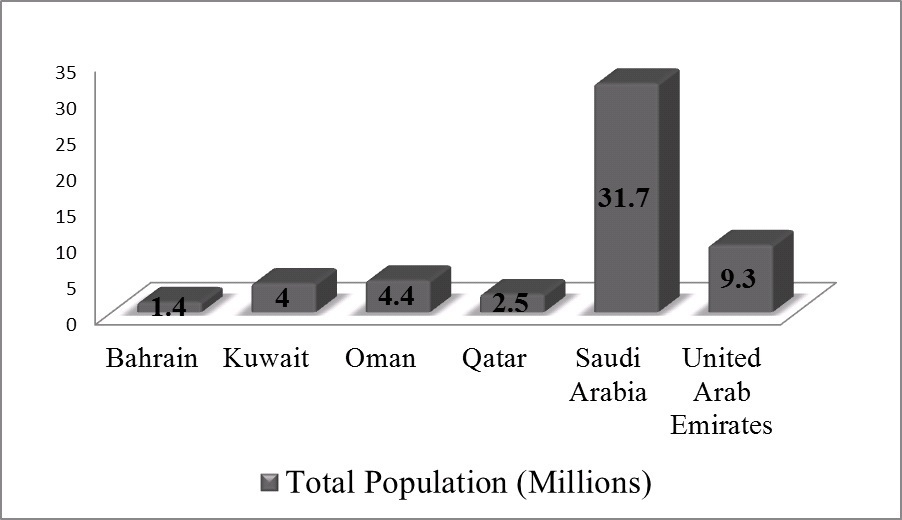
Total population of Gulf Cooperation Council (GCC) countries (2016). Saudi Arabia is the most populous country among the GCC countries. While, the lowest populated country is Bahrain with 1.4 million population: refer to 'Population reference bureau, World population data sheet' [5].

GCC countries are one of the fastest-growing populations in the world (Figure 1). It is predicted that by 2020 this population will increase by one-third, to the current 53 million people [5]. Moreover, there is a huge influx of expatriates (migrant workers) in GCC countries which constitutes about 48.1% of the total population [6]. The rapid growth, aging population and migration present serious challenges to the health care system including the associated healthcare costs.

Health care sector in the MENA region has seen substantial growth over the past few years with improvement in the quality of health services and infrastructure. Health care expenditure is expected to grow to US$144 billion in 2020 worldwide, with approximately US$69 billion of this health spending is expected from the GCC countries [7]. Increased demand for health care in the GCC is mainly because of the changing demographic and epidemiological trends.

Demographics

Recent industrialization and improvement in health care services have led to an increase in life expectancy in the GCC countries from 62 years in 1970 to 77 years in 2012 and infant mortality rate has decreased from 62 per 1000 live births to less than 9 during the same period [4-6]. Moreover, the leading cause of death from infectious diseases was replaced with a predominance of chronic diseases such as diabetes, hypertension and cardiovascular diseases.

**Figure 2:**
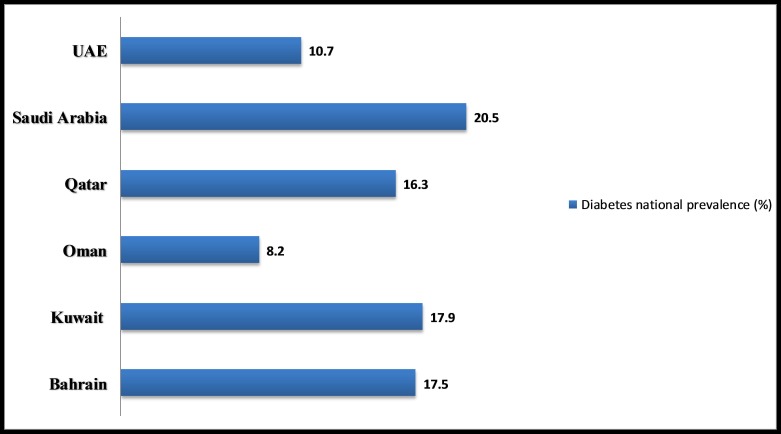
Prevalence of diabetes in Gulf Cooperation Council (GCC). Oman has the lowest prevalence of diabetes (8.2%) among the GCC countries: refer to 'International Diabetes Federation diabetes atlas' [8].

GCC countries rank among the highest in the world on risk factors related to lifestyle ailments such as diabetes, cardiovascular conditions and obesity [8]. According to the International Diabetes Federation, the region has six of the 10 countries in the world with highest diabetes prevalence (Figure 2) [8]. Saudi Arabia has the highest prevalence of diabetes, that is, 20.5% followed by Qatar (16.3%), Kuwait (17.9%), Bahrain (17.5%), UAE (10.7%) and Oman (8.2%) [8].

**Figure 3:**
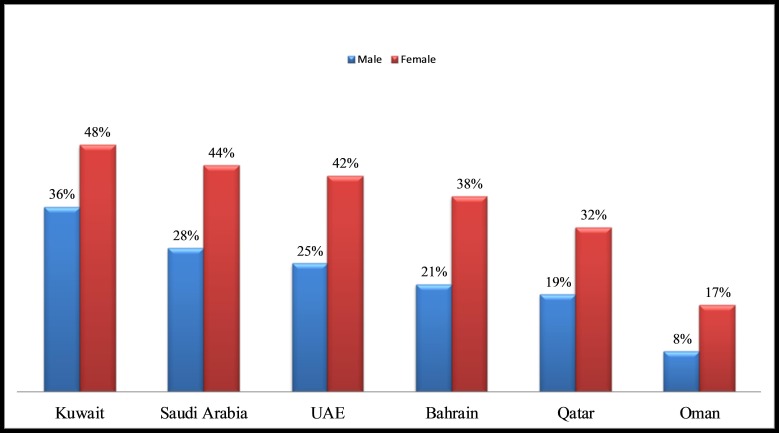
Prevalence of obesity in Gulf Cooperation Council (GCC). The rate of obesity is high for both gender (male: 36%, female: 48%) in Kuwait: refer to 'Global burden disease study results (2013)' [9].

Mortality rate in GCC from NCDs is one of the highest in the world [9]. This is primarily due to the unhealthy lifestyles, including physical inactivity, high caloric diet, lack of focus on health prevention and disease management, weak primary care infrastructure, and inadequate treatment options to manage NCDs and their complications [7, 9]. The obesity rate for GCC nationals stands at an average of 40%, which is one of the highest in the world (Figure 3) [9]. In the coming years, this situation is likely to worsen as the sedentary population in the region ages [9]. 

Initiatives by GCC Governments

As early as 2007, a joint statement of the health ministers in GCC endorsed its commitment to confront the diabetes problem and raised it to the Secretary-General of the Council Secretariat of GCC, to their majesties and highnesses the princes, the leaders of the Cooperation Council States to obtain political commitment to confront this problem. Similarly in 2008, Riyadh Declaration endorsed Gulf charter for Health of the Heart, putting Heart first project [10].

The Supreme Council, being informed of the UN, WHO and the Health Ministers’ Council resolutions, decided to adopt Al-Manama document for control of NCDs (Gulf Plan for Control of NCDs 2011–2020) [11-12]. It directed ministries of health to set executive plans and integrate them in the curative and preventive programs regarding NCDs and to find the best and most effective means for providing services to the citizens. Moreover, the programs should include provision of health education to the community [11-12]. It recommended provision of adequate budgets to combat NCDs.

Recent Initiatives for Prevention and Control of NCDs

Several programs have been initiated by GCC states to sustain progress in health care sector. These include health education in schools (UAE), Health Promotion Council in Bahrain (National Plan for Control of Chronic Diseases), specialized clinics initiatives (Saudi Arabia), NIZWA project for healthy life style (Oman) and the Facts for Life Book (Cooperation Council States) project [13].

There is a need for development and implementation of new approaches in tackling the emerging health care challenges in GCC states. The importance of supporting and promoting the role of primary health care (PHC) in NCDs and proper positioning of the extended medical care should be a significant strategy [13]. A need exists for building human resource capacity and evidence-based guidelines. Strategic directions include networking with Eastern Mediterranean Approaches to NCDs (EMAN) and carrying out the STEP wise approach, by using Gulf plan for health education of NCDs and giving effect to the World Health survey ‘Global Strategy on Diet, Physical Activity and Health’ [14].

There is a need to intensify educational programs regarding healthy life style (diet, exercise) that can assist citizens in modifying lifestyle of the population. Moreover, social initiatives are also needed for periodic health evaluation and smoking control clinics [15-16]. An overall health system reform is the need of the hour. Patient centered care and management require changes from ‘Radar Care’ to ‘continuity of care’ [17].

Challenges and opportunities

The multifaceted nature of health systems across multiple sectors poses challenges in its monitoring and implementation. In this review we will discuss the multiple challenges that exist across the GCC member states for the health care delivery system, using the WHO health system building blocks [18]. The list is long, but the key is to provide opportunities for improvement in middle and long term to facilitate the health services to address the healthcare needs.

Health Service Delivery and Workforce

The current health care system in GCC states functions as radar. The patient appears in the health system and is treated on a “find it and fix it” basis and is discharged, appearing only when next illness episode occurs [19-20]. This healthcare system, (“Radar System”), is not equipped to meet the mounting challenges engendered by the alarming rates of NCDs in GCC. This requires radical changes to the health systems. Problems inhibiting implementation of NCD Programs include lack of risk factor surveillance and absence of reliable mortality data. The solution lies in strengthening primary care and ensuring that services and programs are focused with proactive interventions to promote and prevent risks to health.

Despite the fact that a remarkable increase took place in the number of health care workers there is still a shortage in availability of trained physicians/nurses, especially local professionals in GCC; mainly, due to high turnover rates and retention issues [21]. According to WHO World Statistics Report of 2015, UAE is lagging behind with 31 nursing and midwifery personnel for every 100,000 population [21].

One of the major reasons for this low number can be due to the fact that the GCC healthcare sector depends heavily on expatriate workforce. This poses a challenge for the health care delivery system [22]. Saudi Arabia [78%] and UAE [85%] have the highest percentage of expatriate staff in the health sector [22-23]. Statistics reveal that only 3% of the 23,000 to 25,000 nurses in the country are Emirati [21-23]. Expatriate workforce is expensive and they either prefer to return to their home country after a few years of service or move to the developed countries [21-22].

There is also a challenge in the healthcare delivery system regarding variation in clinician competence, due to the expatriate health workforce, as physicians with different training backgrounds from countries around the world are delivering healthcare in the GCC [23].

Health Care Financing

Healthcare expenditure in the GCC remains low at 3.8% of GDP, below the 10% global average. The UK spends 9.3% of GDP on healthcare, while the US spends the most at 16.2%. By country, total healthcare expenditure as a percentage of GDP in Saudi Arabia and Bahrain is 3.7% which is relatively higher as compared to other GCC member states, i.e., UAE (3.3%), Kuwait (2.7%), Oman (2.3%]) and Qatar (1.9%) [24]. Despite the fact that proportion of GDP spent on health care is low in GCC as compared to other developed countries, a high out of pocket expense on health care is observed in most countries of the region despite the efforts to control it [24]. This healthcare demand and subsequent expenditure is rising sharply in the GCC, with an estimated increase in Compound Annual Growth Rate (CAGR) of 11.4% from 2010 to 2015 [25]. It is predicted that in the next 10 years CAGR (health spending) will further increase [25].

Investment in the private sector may not be the solution. Instead, the governments need to improve the utilization of existing and available resources, invest and strengthen primary care to reduce the costs of hospital care, and invest in public health through collaboration with other ministries outside the health sector [26].

Information and Research

GCC nations have increased investment in healthcare technology. This integration of advanced information technology is expected to make the delivery of healthcare services effective [27]. Qatar has initiated the upgradation of healthcare services by launching an e-health program. Saudi Arabia is digitalizing its hospitals and PHC patients’ medical records through Healthcare Information and Management Systems Society (HIMSS). UAE in 2011 had launched integrated electronic medical record (eMR) system to link public hospitals and clinics across Dubai and the Northern Emirates through health information system – Wareed [28]. According to the UAE Health Ministry, Wareed is expected to facilitate higher accuracy and safety thereby improving the efficiency of the overall public healthcare system [28]. Moreover, Oman MoH had also developed an e-health service by linking identity cards to hospital registration.

These advances act as a catalyst in reducing the unit cost of intervention but will most likely increase overall costs mainly due to increase in demand and misuse of the technologies. This should prompt widespread efforts by stakeholders to cut down costs by promoting more efficient and safe use of resources. Moreover, need exists to conduct studies on cost/benefit analysis of these technologies [28].

Public-Private Partnership

Private health care delivery system is widespread from pharmacies to corporate hospital chains to healthcare insurers. It is indeed a challenge for Universal Health Coverage (UHC) policies to address this complex and diverse healthcare delivery scenario [29].

Established methods are unavailable to evaluate the scope of private health sector in the GCC countries, and policy makers handling and regulating health systems are currently striving to identify the main characteristics of the private sector. Development of metrics to identify the structure and dynamics of private health care service provision in health systems is required across GCC countries. It should include consequences of specific structures, the drivers of change, and levers available to improve efficiency and outcomes [29].

The private sector is an important health-care provider in low and middle-income countries but its role towards UHC is not uniform. The performance of the private sector has been assessed in terms of quality, equity of access, and efficiency. The characteristics of private providers such as size, objectives and technical competence, are important dimensions that impact their performance. Changing the performance of the private sector will require interventions that target the sector as a whole, rather than individual providers [30]. National regulations and independent regulators are needed. In addition, each ministry should develop a clear and specific Health System Performance Assessment (HSPA) framework that measures performances against well-defined targets and holds to account poor performers in both public and private sectors.

Partnership with Consumer and Community

With a rapidly changing health care scenario, a need exists for consumer and community partnership. Positive interaction with the consumer and increased community participation is favorable in meeting challenges. As individuals like to be engaged in healthcare-related issues, both private and public sectors need to be involved with the consumer and the community [31].

Public Health and Clinical Leadership

Health care organizations face challenges to promote the culture that continuously improves the quality of services, patient safety and compassion in health care. A system is needed that can integrate the components and functions of primary and public health care; this can only be achieved through leadership development. Leadership has to provide direction, alignment and commitment within teams delivering health care services [12, 14].

Direction from leadership promotes consensus among team members towards meeting the targets set for health care delivery services and ensures its consistency with vision and values of an organization. Every individual in the organization should be committed towards the success of the organization as a whole, rather than focusing only on individual success in isolation [14].

There is also a clear, compelling and urgent need for leadership cooperation across boundaries and within organizations. Health care has to be delivered increasingly by an interdependent network of organizations. This requires leaders to work together, prioritizing patient care rather than the success of their component. That means leaders are required to work collectively building a cooperative, integrative leadership culture, in fact collective leadership at the system level [32].

Healthcare organizations seeking to invest in their leaders should align organizational needs, system-wide leadership capability and individual leader development. To close the gap between organizations and individual leaders will need a firm understanding of the skills and behaviors required to be effective in each area [32].

Strategic Vision

Multiple state-funded and private providers have raised questions about the strategic directions of leadership in healthcare in GCC. Effective strategic vision requires the attention of policy makers and stakeholders in the region [33, 25]. It is required for designing the health care services in flexible ways to address the needs of the population [33]. Furthermore, leaders should explore new approaches in delivering health care through various stakeholders, who may have different interests and objectives.

Universal Health Coverage

The WHO mandate of UHC ensures delivery of essential health care services across all strata of society without causing financial hardship to health care recipients [29-30]. WHO-EMRO has developed a framework to work on advancing UHC in the EMR based on the following domains:

Developing vision and strategy for UHC Assessing financial risk protection Expansion of the coverage of needing health services Ensuring population eligibility, entitlement and actual coverage

In GCC states, emerging health care challenges are hampering efforts towards ensuring UHC across the region [29-30]. Among several measures, mandatory national (social) health insurance is being considered to ensure UHC across the region [30]. It is imperative for the member states to take more direct responsibility for financial protections for their citizens and expatriates.

Quality of Services and Patient Safety

The WHO has placed particular emphasis on patient safety in health care delivery system [22]. It includes all the health care disciplines and requires a comprehensive multifaceted approach in identifying and managing any actual or potential risks to patient safety in individual services, while finding long-term solutions for the system as a whole.

The objective for implementing Quality Improvement and Patient Safety Programs in GCC states includes improvement in quality of health care delivered, strengthening of community confidence in its health care institutions, reducing unnecessary costs, increasing efficiency, providing credentials for education, protection against lawsuits and facilitating acceptance by and provision of funds from third-party payers [34].

The Jeddah Declaration on patient safety [35] is considered a milestone in promoting and ensuring patient safety across GCC states. It requested countries to initiate their respective national programs on patient safety at all levels including the leadership and national policy makers.

In an attempt to improve patient safety, the health authority of Abu Dhabi (Haad) has implemented the following codes: Providing effective care to patient, having a patient-centered approach, improvement in patient waiting time and also to exercise equity that is to provide care regardless of patients’ gender, ethnicity and socioeconomic status [36].

It is mandatory that organizations remain vigilant to patient safety matters, to avoid any financial and reputational damage. Stakeholders should assess potential deficiencies, define their security and privacy requirements and develop appropriate security programs [1, 9]. One of the most challenging aspects of patients’ safety and quality of service in the GCC is due to the variations in workforce, as the majority of the health care professionals in GCC are from different countries with diverse cultures and training backgrounds.

Recommendations

Short-Term Programs

Short-term programs could be promptly designed and implemented by GCC governments. This will not only help to strengthen the current health care system but will also reduce the modifiable and non-modifiable primary risk factors responsible for alarming rates of NCDs in GCC. There are three domains in which governments can make immediate changes: financial incentives, regulations and health screening.

Financial Incentives

The government can impose increased taxes on unhealthy products such as fast food, soft drinks, and cigarettes. They can provide subsidies on healthy food items such as fruits and vegetables.

Regulations

Adopting and enforcing rules and regulations which limit the availability and promotion of unhealthy products. Awareness programs should be launched in schools and primary care clinics regarding healthy life style including physical activity.

Health Screening

National screening programs to identify at-risk groups (obese and elderly patients) and to ensure early detection of NCDs can be implemented.

Long-Term Programs

Stakeholders should design and launch long-term programs to ensure sustainability of services. Long-term programs are basically designed to focus on behavioral change at individual and at the level of the healthcare system. These programs should mainly cater the youth and adult population.

Youth

The aim of these programs should be to educate teachers and parents about risks of NCDs and importance of healthy lifestyles. These programs can shape the behavior of the children within schools and in their communities.

Adults

Programs aimed at primary care prevention and treatment modalities should be implemented for at-risk/NCDs patients so that, they are better able to manage their conditions and to reduce the impact and complications of NCDs.

## Conclusions

Health care delivery system in GCC member states is facing enormous challenges in meeting the increasing health care demands. These challenges require improvement in health services across GCC countries. Developing and implementing strategic plans to improve the delivery system in GCC states is recommended on an urgent basis. Such plans should focus on shifting the service modality from hospital to primary care, from reactive to proactive services and from radar care to continuity of care. Leadership development in public health is also required to face the challenges and capture the enormous opportunities to improve the health of the GCC population.
